# Ensemble approach for potential habitat mapping of invasive *Prosopis *spp*.* in Turkana, Kenya

**DOI:** 10.1002/ece3.4649

**Published:** 2018-11-21

**Authors:** Wai‐Tim Ng, Alexsandro Cândido de Oliveira Silva, Purity Rima, Clement Atzberger, Markus Immitzer

**Affiliations:** ^1^ Institute for Surveying, Remote Sensing and Land Information (IVFL) University of Natural Resources and Life Sciences (BOKU) Vienna Austria; ^2^ Image Processing Division (DPI) National Institute for Space Research (INPE), Avenida dos Astronautas São José dos Campos Brazil; ^3^ Kenya Forestry Research Institute (KEFRI) Baringo Sub Centre Marigat Kenya; ^4^ Faculty of Arts and Humanities Department of Geography, Chuka University Chuka Kenya

**Keywords:** ensemble modeling, expert knowledge, invasive alien species, *Prosopis*, species distribution modeling

## Abstract

**Aim:**

*Prosopis *spp. are an invasive alien plant species native to the Americas and well adapted to thrive in arid environments. In Kenya, several remote‐sensing studies conclude that the genus is well established throughout the country and is rapidly invading new areas. This research aims to model the potential habitat of *Prosopis *spp*.* by using an ensemble model consisting of four species distribution models. Furthermore, environmental and expert knowledge‐based variables are assessed.

**Location:**

Turkana County, Kenya.

**Methods:**

We collected and assessed a large number of environmental and expert knowledge‐based variables through variable correlation, collinearity, and bias tests. The variables were used for an ensemble model consisting of four species distribution models: (a) logistic regression, (b) maximum entropy, (c) random forest, and (d) Bayesian networks. The models were evaluated through a block cross‐validation providing statistical measures.

**Results:**

The best predictors for *Prosopis* spp*.* habitat are distance from water and built‐up areas, soil type, elevation, lithology, and temperature seasonality. All species distribution models achieved high accuracies while the ensemble model achieved the highest scores. Highly and moderately suitable *Prosopis* spp*.* habitat covers 6% and 9% of the study area, respectively.

**Main conclusions:**

Both ensemble and individual models predict a high risk of continued invasion, confirming local observations and conceptions. Findings are valuable to stakeholders for managing invaded area, protecting areas at risk, and to raise awareness.

## 1 INTRODUCTION

Invasive alien species (IAS) are key drivers of global change and have extensive adverse ecological (i.e., ecosystems and biodiversity), economic (i.e., agriculture and forestry), and social (i.e., allergies and toxins) impacts (Pimentel et al., 2001). IAS cause major damages and losses, adding up to an estimate of $120 billion per year in the United States alone (Pimentel, Zuniga, & Morrison, 2005). Wise, Wilgen, and Maitre (2012) report exceeding control costs of >US$9.5 million/year for the Northern Cape Province, South Africa. Kenya has experienced a number of biological invasions over the past decades, some of which with significant socioeconomic consequences (Lyons & Miller, 1999).

*Prosopis* is a genus of woody tree species deliberately introduced to Kenya by a number of NGO’s (i.e., FAO, NORAD). Many of these environments are vulnerable to vegetation loss and desertification due to increasing population pressure and extreme weather events triggered by climate change. Therefore, *Prosopis *spp. were propagated to rehabilitate these degraded arid environments as they are well adapted to thrive in arid and semi‐arid environments. In the 1980s, a selection of different members of the *Prosopis *genus (i.e., *P. juliflora*, *P. pallida*, *P. chilensis*) was introduced at several test sites throughout Kenya (Choge, Ngunjiri, Kuria, Busaka, & Muthondeki, 2002). This led to a hybridization process described by Pasiecznik et al. (2001) as the *P. juliflora–P. pallida* complex. The hybrid species is well adapted to its new environment and is nowadays regarded as an aggressive invader.

Prosopis spp*.* are ranked as the second worst invasive alien plant taxon in South Africa (Henderson, 2007) and can be found on the World Conservation Unions 100 list of the “world’s worst invasive alien species” (Lowe, Browne, Boudjelas, & Poorter, 2000). In East Africa, *Prosopis* spp*.* have become increasingly abundant (Meroni et al., 2016; Ng, Meroni, et al., 2016b; Rembold, Leonardi, Ng, Gadain, & Meroni, 2015). Besides, reducing biodiversity and replacing endemic species its negative impacts include (a) altering the groundwater tables (Fourie, Mbatha, Verster, & Dyk, 2007), (b) invading communal pastoral lands (Shackleton, Maitre, Wilgen, & Richardson, 2015), (c) its thorns causing injuries to humans and cattle (Van de Giessen, 2011), and (d) to puncturing tires (Swallow & Mwangi, 2008).

In Turkana County, Kenya *Prosopis *spp*.* has become omnipresent (Ng, Immitzer, et al., 2016a). It is crucial to understand its invasion dynamics to effectively negate the adverse impacts and to build analytical frameworks to manage priority areas, that is, early detection of outbreaks and eradication efforts (Schachtschneider & February, 2013; Shackleton, Maitre, Pasiecznik, & Richardson, 2014). In 2017, the Ethiopia ministry of livestock and fisheries published the national strategy on *P. juliflora* management (MOLF, 2017). The reports state that early detection is vital, as removal becomes increasingly challenging after establishment, involving high costs of mechanical and chemical control, combined with the needed repetition due to the presence of seeds in the seedbank, that is, seed viability is 10–15 years (Pasiecznik & Felker, 1992). Despite its abundance and experienced negative impacts (Ng et al., 2017), a national strategy for combating *Prosopis *spp*.* invasion in Kenya is still absent.

Species distribution modeling (SDM) has demonstrated its value in a wide range of applications (Elith & Leathwick, 2009). SDMs can be applied to IAS if two core assumptions are considered: (a) IAS are generally not in equilibrium with their environment and (b) niche quantification and transferability in space and time are limited (Gallien, Douzet, Pratte, Zimmermann, & Thuiller, 2012). Establishing whether IAS operate within the constraints of conservative ecological niches, or whether niche shifts occur as part of the invasion process, is indispensable to identifying and anticipating potential areas of invasion (Araújo & Pearson, 2005). Nonetheless, SDM is an important tool to understand invasion process as it can predict encroachment (Uden, Allen, Angeler, Corral, & Fricke, 2015) or habitat at risk (Ward, 2007).

Shackleton et al. (2014) describe the current and potential global distribution of *Prosopis *spp. and identified many climatically suitable areas which have currently no records of *Prosopis *spp. However, there is little information available on *Prosopis *spp. distribution modeling (Abbas et al., 2016; Irfan‐Ullah, Sharma, & Davande, 2006). Wakie, Evangelista, Jarnevich, and Laituri (2014) applied a SDM for predicting the current and potential distribution for *P. juliflora* in the Afar region in Ethiopia. The study applied a maximum entropy model and utilized several satellite‐derived parameters to map the potential distribution of *P. juliflora*. Remotely derived parameters were enhanced vegetation indices (EVIs) and normalized difference vegetation indices (NDVIs) of moderate‐resolution imaging spectroradiometer (MODIS) time series, WorldClim bioclimatic variables, and shuttle radar topography mission (SRTM) data.

Evangelista et al. (2008) applied five different SDMs to model potential distribution of generalist and specialist invasive plant species in the Grand Staircase‐Escalante National Monument, located in south‐central Utah, USA. The authors conclude that most of the tested SDMs behave similarly, however, generalist species, such as *Prosopis*, are more difficult to predict, while specialist species and their specific habitat requirements are more easily defined by predictive models. Stohlgren et al. (2010) proposed an ensemble model (EM) for mapping invasive species and compared five individual models against an EM for four invasive plant species in four different study sites. The two evaluation datasets (reserved test data and field surveys) indicate that individual models vary in their performance. The ensemble approach, on the other hand, adds substantial robustness and consistency of performance among the different species and study sites. The authors point out that the EM approach can be particularly useful to model recently introduced invasive alien species as these have not yet spread to all suitable niches.

The limited number of studies dedicated to *Prosopis *spp*.* habitat modeling and the urgency to create a framework to effectively manage *Prosopis *spp*.* in Kenya indicates a clear need to accurately determine areas at risk of invasion. Therefore, we model potential *Prosopis *spp*.* habitat by applying an ensemble approach combining four SDMs and assess environmental and expert knowledge‐based variables for Turkana County, Kenya.

## 2 MATERIAL AND METHODS

### 2.1 Test species: *Prosopis *spp.

**Figure 1:**
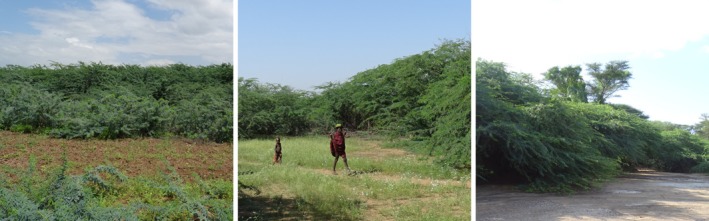
*Prosopis *spp. invading: (left) farmland with young plants emerging on the foreground, (center) pastoral land, and (right) near ephemeral rivers

The *Prosopis *spp. are a prolific woody tree species native to the Americas and characterized by their adaptive traits and propagation strategy. *Prosopis *spp. are capable of growing a deep taproot, which can extend to extreme depths in search for the water table, thus, being less dependent on often unpredictable precipitation (Shiferaw, Teketay, Nemomissa, & Assefa, 2004). The species dispersal strategy utilizes both biotic and abiotic processes (Harding & Bate, 1991). Its leaves are nonpalatable by most herbivores, while the pods are high in sugar content and eaten by many animals (e.g., goats, cattle, baboons), thus spreading seeds and instigating new invasions (Pasiecznik et al., 2001). In Kenya, native plant communities are under anthropogenic pressure due to overutilization, that is, fuelwood collection and livestock browsing (Groot & Hall, 1989), resulting in *Prosopis *spp. having a competitive advantage over the endemic vegetation. *Prosopis *spp*.* can be found throughout the study area at many stages of invasion and appears at high concentrations near farmland (Figure 1, left), pastoral land (Figure 1, center), and periodically dry rivers (Figure 1, right).

### 2.2 Study area

**Figure 2:**
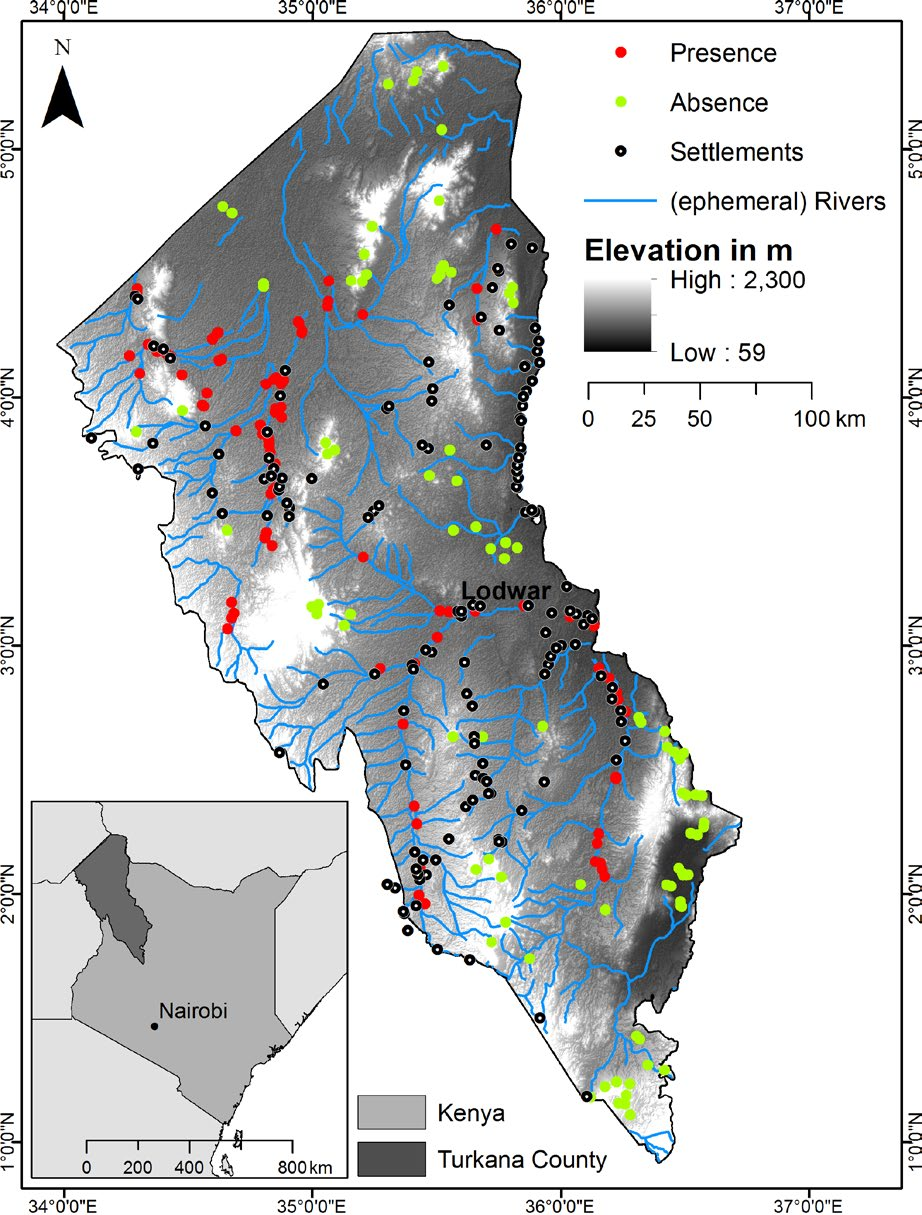
The study area of Turkana, Kenya, and the reference data set (red: presence; green: absence). Data are displayed with the ASTER GDEM along with ephemeral rivers (blue) and settlements (transparent circle)

The Turkana County in Kenya was selected as study area because of the high prevalence of *Prosopis *spp*.* (Ng, Immitzer, et al., 2016a). The study area is located in the Rift Valley of northern Kenya (Figure 2) between latitudes 01°00′N and 05°28′N and longitude 34°02′E and 36°43′E, covering 68,680 km^2^. The County borders Ethiopia in the North, South Sudan in the Northwest, and Uganda in the West. The eastern border consists of Lake Turkana, which is the world’s largest permanent alkaline desert lake. The northern border, called the Ilemi triangle located between Kenya, South Sudan, and Ethiopia, is disputed and claimed by both South Sudan and Kenya.

Turkana is dominated by a “hot desert climate” (Köppen‐Geiger BWh) and to a lesser extent “hot semi‐arid climate” (BSh). The average annual precipitation is 250 mm and rainfall is often unpredictable, with some regions receiving no precipitation during an entire year. The western part is mountainous (1,500–1800 m a.s.l.), while most of the county consists of low‐lying plains (900 m a.s.l.) with perennial rivers draining into Lake Turkana (east) at 360 m a.s.l. (Figure 2). The soils are mostly of volcanic origin, and sediments are relatively low in organic matter. Vegetation is dominated by scattered *Vachellia* bush with *Maerua *spp*.* and *Vachellia tortilis* along the river banks.

### 2.3 Presence and absence data

For training the models, we used in situ collected reference data (Figure 2), acquired during the dry season of 2016 (February) in collaboration with the National Drought Management Authority (NDMA), Kenya. This data set consists of 91 absences and 92 presence points, precisely located with help of GPS. To insure a well‐stratified reference data set (Figure 2, presence [red] and absence [green]), we supplemented the in situ collected data with photo‐interpreted points, made by experienced photo‐interpreters on very high‐resolution Google Earth data. No presence data from the native range (i.e., South and Central America) were used.

### 2.4 Expert knowledge

*Prosopis *spp*.* are a generalist species thus complicating the variable selection process (Evangelista et al., 2008). To achieve improved modeling accuracy, we, therefore, incorporated environmental variables based on expert knowledge (Fourcade, 2016; Mainali et al., 2015). These were derived from fieldwork, mapping efforts, and literature review. Utilizing expert knowledge for the selection and parametrization of input variables allows us to improve the SDM results. The inclusion of expert knowledge is key as the hybrid species’ distribution and habitat are not fully described in the literature. However, Fernández and Hamilton (2015) highlight that ecological niche in the native range is often a poor predictor for the invaded range.

We supplemented our expertise of the genus’ preferred environment with data on the parent species (*P. julifloria *and *P.* *pallida*) habitat provided by Pasiecznik et al. (2001). *Prosopis *spp*.* grow in its native range at elevations between 0 and 1,500 m a.s.l. The presence and depth of the water table are a decisive factor in the distribution, size, and growth, while poor water availability and soil fertility do not limit growth. *Prosopis *spp*.* thrive in almost all soil types ranging from pure sands to heavy clays, and the species perform well on saline (18,000 mg NaCl/L) and alkaline (pH 11) soils (Singh, 1996). However, soil depth is important and thin soils are unsuitable (NAS, 1980). Abundant rainfall is a limiting factor as the species is less common in regions with more than 1,000 mm of mean annual rainfall (NAS, 1980). The species prefers mean annual temperatures above 20°C with an optimal between 20 and 30°C. It has a tolerance for day‐time shade temperatures of 50°C and soils temperatures in full sunlight as high as 70°C. *Prosopis *spp*.* are hindered by low temperatures and light frost can cause dieback; however, some species can handle frost (Felker, Clark, Nash, Osborn, & Cannell, 1982). This information enables us to make general assumptions about the suitable environmental conditions for *Prosopis *spp.

### 2.5 Environmental variables

The tested environmental and expert knowledge‐based variables are provided in Supporting Information, Table S1. Some variables were subjected to interpretation, such as assigning values for the creation of buffers for the waterways, road network, and built‐up areas. We gradually reduced the number of variables until we had a stable output consisting of variables (a) which contribute to the model accuracy and (b) simultaneously ecologically meaningful. We selected the variables based on three conditions: (a) variable correlation, as determined by the pairwise Pearson and Spearman tests scoring <0.70 (Immitzer, Nopp‐Mayr, & Zohmann, 2014; Sachser et al., 2017); (b) variable collinearity as determined by the variance inflation factor scoring <0.90 (VIF, Zuur, Ieno, & Elphick, 2010); and (c) variable bias, as determined by assessing the outputs and identifying overly dominant variables. The variables were evaluated, and only statistically and ecologically significant predictors were retained.

All variables covered the entire study area. The variables were preprocessed including (a) homogenizing the EPSG projection (i.e., WGS 84/UTM 36 N), (b) resampling to 100 by 100‐m spatial resolution (aggregating and disaggregating), and (c) masking with the study area. The tasks were performed with the “*rgdal*” package (Bivand et al., 2017) in R version 3.4.0 (R Core Team, 2017). The Pearson and Spearman tests were performed with the R package “Hmisc” (Harrel, 2015) and the VIF test was performed in R, according to a script provided by Zuur et al. (2010).

### 2.6 Distribution models

**Figure 3:**
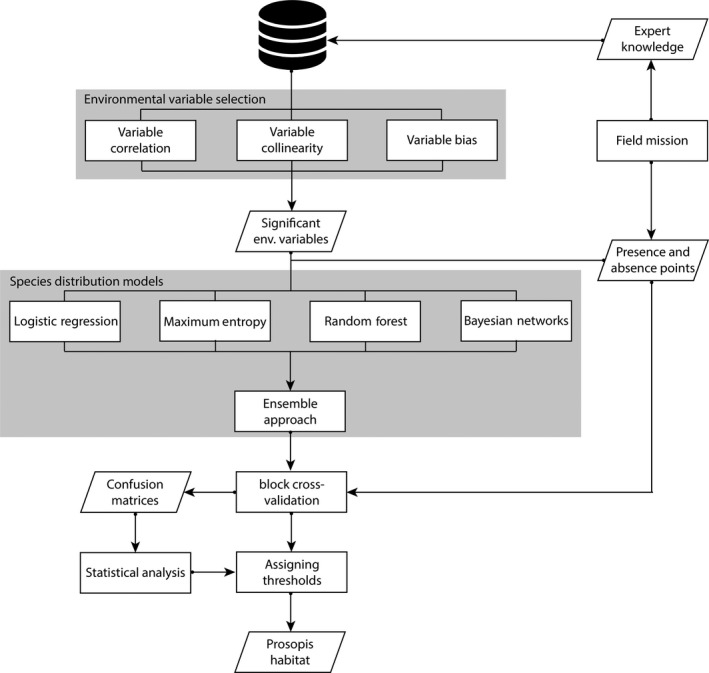
The workflow for predicting *Prosopis *spp*.* habitat. The evaluation (block cross‐validation and threshold assignment) was also performed on the individual models

In total, four distribution models were studied. Three models, logistic regression, random forest, and Bayesian networks, used an identical set of presence and absence points for building the model, while maximum entropy automatically generated background points or pseudo‐absence data (Figure 3). We used the default value of 10,000 background points and the same presence points used for training the other models.

### 2.7 Ensemble modeling

Species distribution modelings have applications in a wide range of disciplines (Franklin, 2010) and are considered an acknowledged tool for predicting invasive alien species distribution and habitat (Dlamini, 2016; Duscher & Nopp‐Mayr, 2017; Keith & Spring, 2013; Lemke & Brown, 2012; Masocha & Dube, 2017). An ensemble model (EM) combines the strengths of several SDM approaches while minimizing the weakness of any particular model (Capinha & Anastácio, 2011; Stohlgren et al., 2010). The four SDMs used to create the ensemble model are logistic regression (LR), MaxEnt (ME), random forest (RF), and Bayesian networks (BN). All SDMs used identical sets of environmental variables and reference data. An overview of the SDMs can be found in the Supporting Information, Table S2.

### 2.8 Model evaluation

From the output of each SDM, one can generate a binary map with the *Prosopis *spp*.* presence and absence classes by slicing the output using a probability threshold. Pixels with values below this threshold are labeled as absence while pixels with values above it are labeled as presence. To determine the best probability threshold for each SDM, we used the kappa index maximization approach (Guisan, Theurillat, & Kienast, 1998). Following this approach, the best threshold is the one that produces a binary map with the highest kappa index when compared with the entire reference dataset (91 absences and 92 presence points). For each SDM, we generated statistical measures such as (a) sensitivity; (b) specificity; (c) true skill statistics; (d) overall accuracy; (e) Cohen's kappa; and (f) Area under the receiver operating characteristic (ROC) curve. These are reported in Supporting Information, Table S3. To generate an accurate habitat suitability map, all the pixels with values below the best probability threshold were classified as “nonsuitable” area while all the pixels with values above it were divided into three classes: “low, “moderate,” and “high habitat suitability.”

We also evaluated each individual SDM output by applying a block cross‐validation (El‐Gabbas & Dormann, 2017; Guevara, Gerstner, Kass, & Anderson, 2017; Roberts et al., 2017). Cross‐validation has the advantage that it optimizes the limited amount of reference data and minimizes the risk of over‐fitting. Although our reference points are well distributed over the study area, the presence/absence points are sometimes clustered. This leads to an unbalanced validation data sets, with blocks entirely lacking either presence or absence points. To counter this imbalance, we created nongridded irregular shaped blocks as described by Roberts et al. (2017). This insured that each of the blocks (*n* = 12) has a proportional amount of presence and absence data. Afterward, confusion matrices were created from the comparison between testing omitted points and the binary map, which was produced according to the calculated probability threshold. This was repeated 12 times until all reference points were used for evaluation. Finally, all 12 confusion matrices were merged into a single one to compute the (a‐e) statistical measures for the block cross‐validation.

## 3 RESULTS

### 3.1 Variable importance and selection

Based on the expert knowledge and the conducted tests (i.e., variable importance, collinearity, and bias, Supporting Information, Table S4), we determined that the following eight features were best suited for modeling *Prosopis *spp*.* habitat: (a) distance to water (Dist. from water: HydroSHEDS); (b) distance to built‐up (Dist. from urban: DLR‐GUF); (c) distance to roads (Dist. from roads: OSM); (d) lithology (SOTWIS); (e) soil type (SOTWIS); (f) landform (SOTWIS); (g) elevation (ASTER GDEM); and (h) temperature seasonality (Temp. seasonality: BIO4).

**Figure 4:**
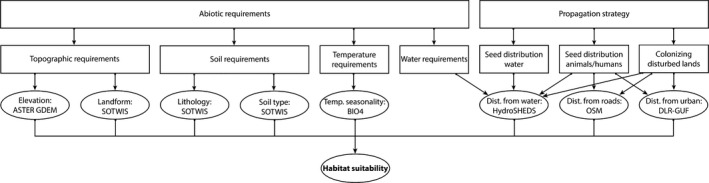
A Directed acyclic graph (DAG) representing habitat suitability of *Prosopis *spp. The rectangular nodes proved the condition/justification and underlying process for using a variable. The full variable description can be found in Table S1

These selected variables were applied to build the SDMs. In addition to the variables listed in Supporting Information Table S1, we performed tests with multispectral data from Sentinel‐2 satellite and NDVI time series from smoothed and gap‐filled Landsat data (Vuolo, Ng, & Atzberger, 2017). We found that these satellite data did not positively contribute to predict the potential habitat. We therefore did not include these variables in the models. We also fitted the selected variables into a directed acyclic graph (DAG, Figure 4). This represents the probabilistic relationship among the variables, and the conditional (in)dependency is an essential function of Bayesian networks. For each node in the structure, there is a conditional‐probability function that relates the node to its immediate parent. To improve the rational, the relationships between the nodes were added, displaying the underlying process influencing habitat suitability.

### 3.2 Model outputs and evaluation

Table 1 shows the statistical measures of the combined block cross‐validation for the SDMs and EM. The probability threshold for each model was quite variable, from 0.165 for the MaxEnt to 0.635 for the Bayesian Network. All statistical results indicate high modeling performance. RF and the EM performed slightly better compared to the other SDMs.

**Table 1:** Accuracy assessment of the block cross‐validation modeling results

	LR	ME	RF	BN	EM
Probability threshold	0.205	0.165	0.560	0.635	0.425
Sensitivity	0.978	0.912	0.989	0.901	0.989
Specificity	0.859	0.967	1.000	0.946	0.989
True skill statistic	0.837	0.879	0.989	0.847	0.978
Overall accuracy	0.918	0.940	0.995	0.923	0.989
Kappa index	0.836	0.880	0.989	0.847	0.978

Note

BN: Bayesian Network; EM: Ensemble model; LR: logistic regression; ME: MaxEnt; RF: Random Forest.

**Figure 5:**
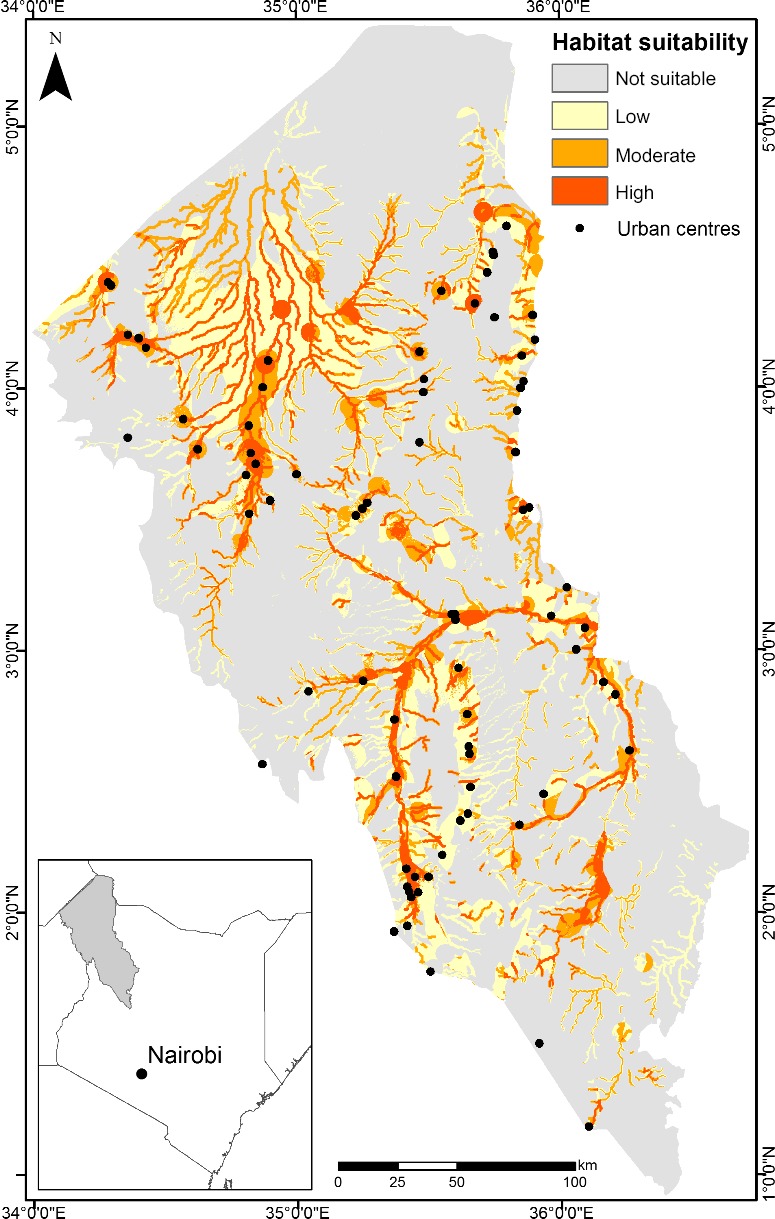
The *Prosopis *spp*.* habitat suitability map of the ensemble model. The pixels with values above the probability threshold of 0.43 were divided into “low” (yellow), “moderate” (orange), and “high” (red) suitable habitat

The habitat suitability map of the ensemble model, displayed in Figure 5, was calculated by averaging the four SDMs outputs. The probability threshold was set at 0.43 (Table 1, EM). Pixels with a value below this threshold are regarded as not suitable *Prosopis *spp*.* habitat. There remaining values were divided into three classes: “low” (0.43–0.62), “moderate” (0.62–0.81), and “high” (0.81–1.0) *Prosopis *spp*.* suitability. The habitat suitability maps of the single models can be found at Supporting Information, Figure S1.

The area of each habitat suitability class is shown in Table 2. The majority of the study area (69%) can be considered as not suitable habitat for *Prosopis *spp. However, relatively large areas of high and moderate suitable habitat are present, covering, respectively, 420,450, and 596,552 ha, together roughly 15% of the total area. An additional 16% of the area is assigned a low suitability.

**Table 2:** Area of each habitat suitability class of the ensemble model output. The pixels were grouped in not suitable, low, moderate, and high habitat suitability

Habitat suitability	ha	Area (%)
Not suitable	4,704,426.947	69
Low	1,100,733.03	16
Moderate	596,551.962	9
High	421,450.12	6

## 4 DISCUSSION

### 4.1 Environmental variables

The inclusion of expert knowledge‐based variables positively contributed to predicting potential habitat as these variables were consequently selected during the variable assessment process. Our observations are in line with Kuhnert, Martin, and Griffiths (2010) who proposed guidelines for expert knowledge‐based modeling and stated that expert knowledge can increase the precision of models and facilitate informed decision making in a cost‐effective manner. Bazzichetto et al. (2018) determined that the percentage of artificial land and distance from roads exhibit a significant relationship with the occurrence of *Carpobrotus* sp. Furthermore, high‐quality environmental variables (i.e., land use data, soil data) describing species habitat are fairly scarce and produced by multiple research institutes using different methods (e.g., measurements, interpolations) and standards (e.g., projections, spatial resolutions). Certainly, our modeling would have benefited from additional variables which were unfortunately not available such as groundwater table, soil depth, or climate data at a higher spatial resolution (Lowen, McKindsey, Therriault, & DiBacco, 2016). The bioclimatic variables (Temp. seasonality: BIO4) had prevailing low spatial resolution and are possibly not suitable for smaller test sites. Additionally, the quality of these data can be affected by the scarcity and reliability of the weather stations in East Africa (Van Gils, Westinga, Carafa, Antonucci, & Ciaschetti, 2014).

### 4.2 Ensemble model and evaluation

The models (i.e., EM and four SDMs) provide highly accurate and comparable results, as shown through the block cross‐validation. The statistical measures rank RF best, scoring the highest accuracies followed by the EM. Shiferaw, Bewket, and Eckert (2018) achieved similar results when mapping fractional *P. juliflora* cover in Afar, Ethiopia, and concluded that random forest performed best closely followed by the EM. In our opinion, the EM presents a good compromise mitigating the uncertainties provided by modeling the potential habitat of invasive species. By not relying on a single model, or their potential flaws, the ensemble approach adds considerable robustness and consistency, thereby confirming the observations made by Stohlgren et al. (2010).

### 4.3 Potential distribution and invasion pattern

The EM output was in agreement with our field observations and in correspondence with the *Prosopis *spp*.* cover for the Tarach basin, Turkana County, Kenya mapped in 2016 (Ng, Immitzer, et al., 2016a). This study applied remote sensing for detecting the *Prosopis *spp.*,* cover in 2016, using Sentinel‐2 satellite data and in situ observations. Their results indicated that *Prosopis *spp*.*—classified into dense, sparse, and mixed classes—covered, respectively, 1.53%, 4.61%, and 1.77% of the total land cover. If we consider that the amount of highly to moderately suitable habitat accounts for 15% of the study area, we can expect the area covered by *Prosopis *spp*. *in 2016 to double in the future, not taking into consideration projected climate models. Our results also confirmed that *Prosopis* spp. are mainly found near the ephemeral rivers (Schachtschneider & February, 2013; Shackleton et al., 2015) and settlements (Meroni et al., 2016; Ng et al., 2017). It is clear that these areas provide conditions which are very suitable for *Prosopis *spp*.*, as they provide three key components: water, anthropogenic disturbance, and distribution of seeds. The higher water availability promotes plant growth and, thus, supports more biomass (i.e., lush vegetation or crop production). This is highlighted by the importance of the distance to water variable to the models. Higher population density, which is otherwise very low throughout the study area, leads to increased pressure on native plant communities, through the collection of fuelwood and clearing vegetation for crop production. Higher population density also results in increased livestock numbers, causing additional stress on native vegetation through grazing by cattle and browsing by goats. The distance to settlements and the road network variables are illustrative to this process. Finally, livestock is an important propagator of *Prosopis* seeds and driver of the invasion. The presence of livestock can be explained by three variables: distance to water, roads, and settlements. Together with the strong spatial correlation between settlements and water presence, these expert knowledge‐based variables are good indicators for highly suitable *Prosopis *spp*.* habitat and invasion risk, as depicted in the model output (Figure 5).

## 5 CONCLUSION

Our study determined the potential habitat of *Prosopis *spp*.* in Turkana, Kenya, using an ensemble approach incorporating four different species distribution models. At the same time, environmental and expert knowledge‐based variables were assessed. *Prosopis *spp*.* have not yet fully occupied their entire ecological niche of their respective new ecosystem, give the relatively short amount of time since the species has established itself, this is also strengthened by the disparity between the distribution in 2016 and the potential habitat. The lack of equilibrium, and the fact that one has to deal with a hybridized species, makes modeling efforts particularly challenging. Nonetheless, the species is causing severe negative impact by altering biodiversity (i.e., replacing many indigenous species) and economically crippling livelihood activities (i.e., invading croplands and restricting access to water). This warrants immediate action with the aim of eradication. Unfortunately, experiences from the Americas, Africa, and Australia teach us that eradication of *Prosopis* spp. has proven to be extremely difficult or sometimes even impossible. This is largely due to the fast regrowth rate of *Prosopis* spp. from vegetative buds and the viable seeds deposited in the seed bank. The impoverished societies in the developing world have certainly only very limited resources to effectively combat this process. Therefore, while preparing for eradication, we call for better management of invaded high ecologic and economic areas, with special focus on awareness raising and prevention, by protecting not yet infested highly suitable habitat.

## CONFLICT OF INTEREST

None declared.

## AUTHOR CONTRIBUTIONS

W‐TN, CA, and MI involved in conceptualizing and planning experiment; W‐TN, ACOS, and MI performed the modeling and validation; W‐TN, CA, and MI performed the analysis; and all authors wrote the manuscript.

## DATA ACCESSIBILITY

The reference data can be found at the Dryad Digital Repository: 10.5061/dryad.sk4410m.
