# Supplementary figures and images for: Ensemble approach for potential habitat mapping of invasive Prosopis spp. in Turkana, Kenya
Source: Ecol Evol. 2018 Nov 21;8(23):11921–31. doi: 10.1002/ece3.4649 (PMC6303778; doi:10.1002/ece3.4649)

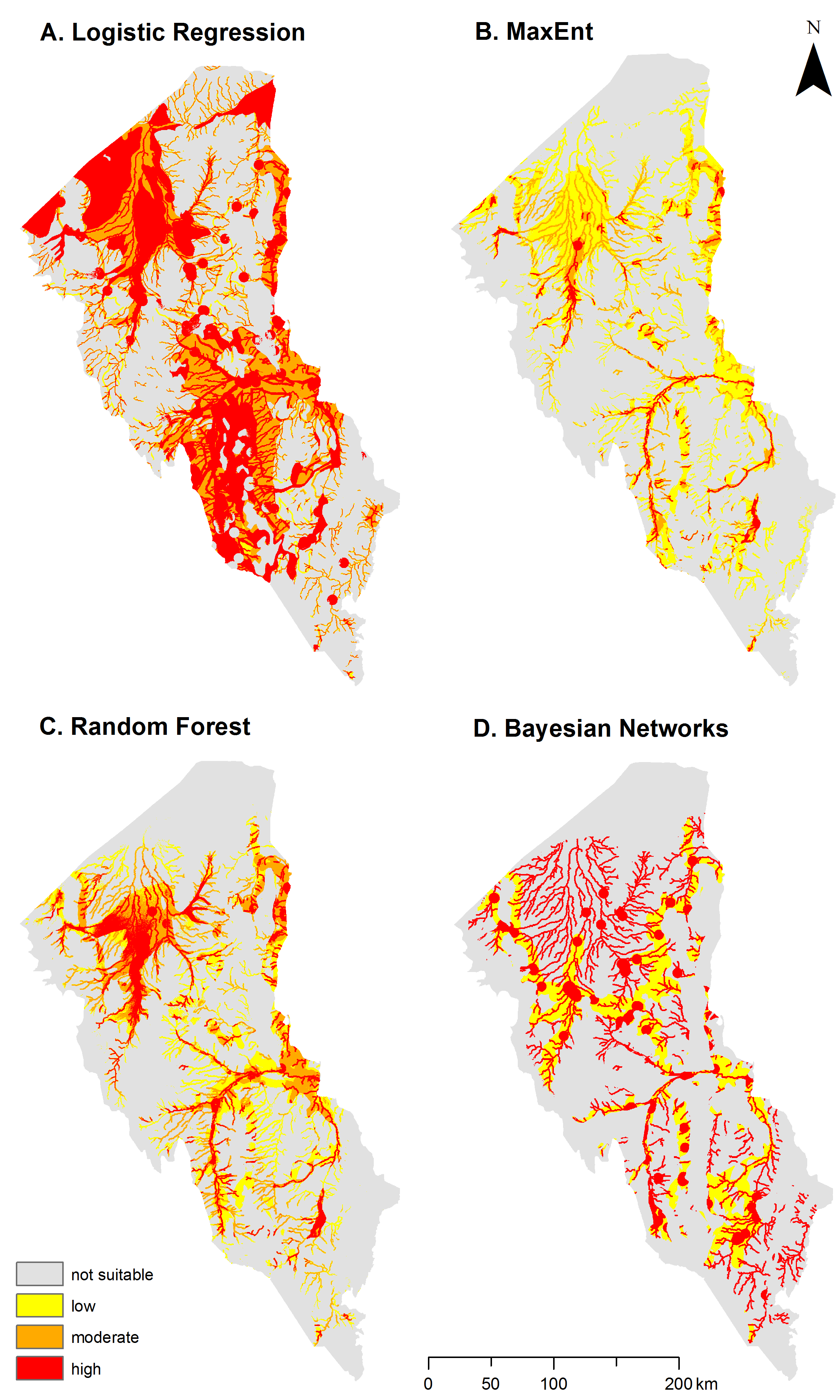

Supplement: Supplementary file 1 [file ECE3-8-11921-s001.png]
