# Supplementary material for: Ensemble approach for potential habitat mapping of invasive Prosopis spp. in Turkana, Kenya
Source: Ecol Evol. 2018 Nov 21;8(23):11921–31. doi: 10.1002/ece3.4649 (PMC6303778; doi:10.1002/ece3.4649)
Supplement: Supplementary file 2 [file ECE3-8-11921-s002.docx]

**Supplementary material**

Table S1. Overview of all tested environmental variables (n=30). Variable descriptions and source are also included.

| **Name** | **Descirption** | **Data source** |
| --- | --- | --- |
| **Elevation** | Elevation a.s.l. | ASTER GDEM 2.0 (Abrams and Hook, 2002) |
| **Aspect** | downslope direction derived from elevation | ASTER GDEM 2.0 (Abrams and Hook, 2002) |
| **Slope** | maximum rate of change derived from elevation | ASTER GDEM 2.0 (Abrams and Hook, 2002) |
| **Dist. water** | Distance to water network | HydroSHEDS (Lehner et al., 2008) |
| **Dist. build-up** | Distance to build-up areas | Global Urban Footprint (GUF) DLR (Esch et al., 2013, 2012) |
| **Dist. road** | Distance to road network | OSM (OpenStreetMap Foundation, 2013) |
| **Soil class** | Dominant soil type | SOTWIS 1.2 – FAO (FAO/IIASA/ISRIC/ISSCAS/JRC, 2012) |
| **Lithology** | Gross physical character of parent material | SOTWIS 1.2 – FAO (FAO/IIASA/ISRIC/ISSCAS/JRC, 2012) |
| **Landform** | Natural terrain features | SOTWIS 1.2 – FAO (FAO/IIASA/ISRIC/ISSCAS/JRC, 2012) |
| **CHIRPS** | Monthly precipitation 2016 | CHIRPS 2.0 (Funk et al., 2015) |
| **TAMSAT** | Monthly precipitation 2014-2016 | TAMSAT (Maidment et al., 2014; Tarnavsky et al., 2014) |
| **BIO1** | Annual Mean Temperature | WorldClim 1.4 (Hijmans et al., 2005) |
| **BIO2** | Mean Diurnal Range (Mean of monthly (max temp - min temp)) | WorldClim 1.4 (Hijmans et al., 2005) |
| **BIO3** | Isothermality (BIO2/BIO7) (* 100) | WorldClim 1.4 (Hijmans et al., 2005) |
| **BIO4** | Temperature Seasonality (standard deviation *100) | WorldClim 1.4 (Hijmans et al., 2005) |
| **BIO5** | Max Temperature of Warmest Month | WorldClim 1.4 (Hijmans et al., 2005) |
| **BIO6** | Min Temperature of Coldest Month | WorldClim 1.4 (Hijmans et al., 2005) |
| **BIO7** | Temperature Annual Range (BIO5-BIO6) | WorldClim 1.4 (Hijmans et al., 2005) |
| **BIO8** | Mean Temperature of Wettest Quarter | WorldClim 1.4 (Hijmans et al., 2005) |
| **BIO9** | Mean Temperature of Driest Quarter | WorldClim 1.4 (Hijmans et al., 2005) |
| **BIO10** | Mean Temperature of Warmest Quarter | WorldClim 1.4 (Hijmans et al., 2005) |
| **BIO11** | Mean Temperature of Coldest Quarter | WorldClim 1.4 (Hijmans et al., 2005) |
| **BIO12** | Annual Precipitation | WorldClim 1.4 (Hijmans et al., 2005) |
| **BIO13** | Precipitation of Wettest Month | WorldClim 1.4 (Hijmans et al., 2005) |
| **BIO14** | Precipitation of Driest Month | WorldClim 1.4 (Hijmans et al., 2005) |
| **BIO15** | Precipitation Seasonality (Coefficient of Variation) | WorldClim 1.4 (Hijmans et al., 2005) |
| **BIO16** | Precipitation of Wettest Quarter | WorldClim 1.4 (Hijmans et al., 2005) |
| **BIO17** | Precipitation of Driest Quarter | WorldClim 1.4 (Hijmans et al., 2005) |
| **BIO18** | Precipitation of Warmest Quarter | WorldClim 1.4 (Hijmans et al., 2005) |
| **BIO19** | Precipitation of Coldest Quarter | WorldClim 1.4 (Hijmans et al., 2005) |

Table S2. Overview of SDMs used in the ensemble approach

| Method | Description | R package | References |
| --- | --- | --- | --- |
| Logistic Regression (LR) | classical approach, envelope model, linear technique | stats  (version 3.5.0) | (McCullagh and Nelder, 1989; Pearce and Ferrier, 2000) |
| Maximum Entropy (ME) | popular approach, machine learning, presence only, generates pseudo-absence data | dismo  (version 1.0-15) | (Hijmans et al., 2011; Jaynes, 1986; Phillips et al., 2004) |
| Random Forest (RF) | machine learning approach, ensemble technique | randomForest (version 4.6-12) | (Breiman, 2001; Mi et al., 2017; Zohmann et al., 2014) |
| Bayesian Networks (BN) | incorporates expert knowledge, computational demanding, considers model uncertainty | e-BayNeRD  (version 1) | (Friedman et al., 1997; Mello et al., 2013; Silva et al., 2014) |

Table S3. Model evaluation and statistical measures

| **Statistical measures** | **Description** | **Source** |
| --- | --- | --- |
| **kappa maximization approach** | Kappa statistic is maximized | (Allouche et al., 2006; Guisan et al., 1998) |
|  |  |  |
| **Sensitivity** | the correctly predicted positive fraction | (Fielding and Bell, 1997) |
|  |  |  |
| **Specificity** | the correctly predicted negative fraction | (Fielding and Bell, 1997) |
|  |  |  |
| **True Skill Statistics** | combine sensitivity and specificity so that both omission and commission errors are accounted for | (Allouche et al., 2006) |
|  |  |  |
| **Overall accuracy** | The proportion of correct predictions in the entire matrix | (Finley, 1884) |
|  |  |  |
| **Kappa index** | a discrete multivariate technique for expressing overall accuracy by comparing two sources of data. How much does the classification differ from a random matrix | (Cohen, 1960) |
|  |  |  |
| **Area under the ROC curve** | estimate the predictive accuracy of distributional models derived from presence–absence species data. Produces continuous probabilities of presence (P), where P and 1 – P represent the degree to which each case is a member of one of the two events | (Guisan and Zimmermann, 2000) |
|  |  |  |

Table S4. Results of the variable selection though variable correlation (Pearson and Spearman tests) and variable collinearity (variance inflation factor scoring) for the eight environmental variables used for modelling potential habitat.

| **Pearson test** | **Dist. road** | **Dist. build-up** | **Dist. water** | **Elevation** | **Landform** | **Lithology** | **Soil class** | **BIO4** |
| --- | --- | --- | --- | --- | --- | --- | --- | --- |
| **Dist. road** | 1 | -0.01 | -0.11 | 0 | -0.05 | 0.01 | 0.14 | -0.19 |
| **Dist. build-up** | -0.01 | 1 | 0.06 | 0.02 | 0.12 | 0.1 | 0.16 | -0.08 |
| **Dist. water** | -0.11 | 0.06 | 1 | -0.09 | -0.14 | -0.06 | 0.14 | -0.05 |
| **Elevation** | 0 | 0.02 | -0.09 | 1 | 0.43 | 0.49 | 0.2 | 0.42 |
| **Landform** | -0.05 | 0.12 | -0.14 | 0.43 | 1 | 0.64 | 0.22 | 0.15 |
| **Lithology** | 0.01 | 0.1 | -0.06 | 0.49 | 0.64 | 1 | 0.46 | 0.08 |
| **Soil class** | 0.14 | 0.16 | 0.14 | 0.2 | 0.22 | 0.46 | 1 | -0.13 |
| **BIO4** | -0.19 | -0.08 | -0.05 | 0.42 | 0.15 | 0.08 | -0.13 | 1 |
| **Spearman test** |  |  |  |  |  |  |  |  |
| **Dist. road** | 1 | 0.18 | -0.05 | 0.12 | -0.07 | 0.1 | 0.19 | -0.22 |
| **Dist. build-up** | 0.18 | 1 | 0.1 | 0.07 | 0.06 | 0.08 | 0.14 | -0.07 |
| **Dist. water** | -0.05 | 0.1 | 1 | -0.06 | -0.15 | -0.08 | 0.1 | -0.04 |
| **Elevation** | 0.12 | 0.07 | -0.06 | 1 | 0.43 | 0.5 | 0.33 | 0.29 |
| **Landform** | -0.07 | 0.06 | -0.15 | 0.43 | 1 | 0.61 | 0.28 | 0.16 |
| **Lithology** | 0.1 | 0.08 | -0.08 | 0.5 | 0.61 | 1 | 0.6 | 0.04 |
| **Soil class** | 0.19 | 0.14 | 0.1 | 0.33 | 0.28 | 0.6 | 1 | -0.06 |
| **BIO4** | -0.22 | -0.07 | -0.04 | 0.29 | 0.16 | 0.04 | -0.06 | 1 |
| **Variance Inflation Factor** |  |  |  |  |  |  |  |  |
| **Dist. road** | 1 | 0.08 | 0.06 | -0.09 | -0.08 | -0.12 | -0.04 | -0.13 |
| **Dist. build-up** | 0.08 | 1 | 0.05 | -0.08 | -0.08 | -0.11 | -0.04 | -0.09 |
| **Dist. water** | 0.06 | 0.05 | 1 | -0.14 | -0.16 | -0.21 | -0.1 | -0.12 |
| **Elevation** | -0.09 | -0.08 | -0.14 | 1 | 0.45 | 0.51 | 0.68 | 0.88 |
| **Landform** | -0.08 | -0.08 | -0.16 | 0.45 | 1 | 0.58 | 0.43 | 0.37 |
| **Lithology** | -0.12 | -0.11 | -0.21 | 0.51 | 0.58 | 1 | 0.58 | 0.41 |
| **Soil class** | -0.04 | -0.04 | -0.1 | 0.68 | 0.43 | 0.58 | 1 | 0.64 |
| **BIO4** | -0.13 | -0.09 | -0.12 | 0.88 | 0.37 | 0.41 | 0.64 | 1 |

**Supplementary Figure**

Figure S1. Model output with probability thresholds (Table 1). The pixels above the threshold were divided into ‘low’ (yellow), ‘moderate’ (orange) and ‘high’ (red) suitable habitat.

**References**

Abrams, M., Hook, S., 2002. ASTER User Handbook Version 2. Jet Propuls. 2003, 135. https://doi.org/10.1017/CBO9781107415324.004

Allouche, O., Tsoar, A., Kadmon, R., 2006. Assessing the accuracy of species distribution models: Prevalence, kappa and the true skill statistic (TSS). J. Appl. Ecol. 43, 1223–1232. https://doi.org/10.1111/j.1365-2664.2006.01214.x

Breiman, L., 2001. Random forests. Mach. Learn. 45, 5–32. https://doi.org/10.1023/A:1010933404324

Cohen, J., 1960. A coefficient of agreement for nominal scales. Educ. Psychol. Meas. 20, 37–46. https://doi.org/10.1177/001316446002000104

Esch, T., Marconcini, M., Felbier, A., Roth, A., Heldens, W., Huber, M., Schwinger, M., Taubenbock, H., Muller, A., Dech, S., 2013. Urban footprint processor-Fully automated processing chain generating settlement masks from global data of the TanDEM-X mission. IEEE Geosci. Remote Sens. Lett. 10, 1617–1621. https://doi.org/10.1109/LGRS.2013.2272953

Esch, T., Taubenböck, H., Roth, A., Heldens, W., Felbier, A., Thiel, M., Schmidt, M., Müller, A., Dech, S., 2012. TanDEM-X mission—new perspectives for the inventory and monitoring of global settlement patterns. J. Appl. Remote Sens. 6, 61701–61702. https://doi.org/10.1117/1.JRS.6.061702

FAO/IIASA/ISRIC/ISSCAS/JRC, 2012. Harmonized World Soil Database (version 1.2).

Fielding, A.H., Bell, J.F., 1997. A review of methods for the assessment of prediction errors in conservation presence / absence models. Environ. Conserv. https://doi.org/10.1017/S0376892997000088

Finley, J.P., 1884. Tornado predictions. Am. Meteorol. J. 1, 85–88.

Friedman, N., Geiger, D., Goldszmidt, M., 1997. Bayesian Network Classifiers. Mach. Learn. 29, 131–163. https://doi.org/10.1023/A:1007465528199

Funk, C., Peterson, P., Landsfeld, M., Pedreros, D., Verdin, J., Shukla, S., Husak, G., Rowland, J., Harrison, L., Hoell, A., Michaelsen, J., 2015. The climate hazards infrared precipitation with stations—a new environmental record for monitoring extremes. Sci. Data 2, 150066. https://doi.org/10.1038/sdata.2015.66

Guisan, A., Theurillat, J.-P., Kienast, F., 1998. Predicting the potential distribution of plant species in an alpine environment. J. Veg. Sci. https://doi.org/10.2307/3237224

Guisan, A., Zimmermann, N.E., 2000. Predictive habitat distribution models in ecology. Ecol. Modell. 135, 147–186. https://doi.org/10.1016/S0304-3800(00)00354-9

Hijmans, R.J., Cameron, S.E., Parra, J.L., Jones, P.G., Jarvis, A., 2005. Very high resolution interpolated climate surfaces for global land areas. Int. J. Climatol. 25, 1965–1978. https://doi.org/10.1002/joc.1276

Hijmans, R.J., Phillips, S., Leathwick, J.R., Elith, J., 2011. Package ‘ dismo .’ October 55. https://doi.org/10.1016/j.jhydrol.2011.07.022.

Jaynes, E.T., 1986. Monkeys, Kangaroos, and N. Maximum-Entropy Bayesian Methods Appl. Stat. 26.

Lehner, B., Verdin, K., Jarvis, A., 2008. New global hydrography derived from spaceborne elevation data. Eos (Washington. DC). 89, 93–94. https://doi.org/10.1029/2008EO100001

Maidment, R.I., Grimes, D., Allan, R.P., Tarnavsky, E., Stringer, M., Hewison, T., Roebeling, R., Black, E., 2014. The 30 year TAMSAT African Rainfall Climatology And Time series (TARCAT) data set. J. Geophys. Res. Atmos. 2014JD021927. https://doi.org/10.1002/2014JD021927

McCullagh, P., Nelder, J.A., 1989. Generalized Linear Models, 2nd edditi. ed. Chapman and Hall, London.

Mello, M.P., Risso, J., Atzberger, C., Aplin, P., Pebesma, E., Vieira, C.A.O., Rudorff, B.F.T., 2013. Bayesian networks for raster data (BayNeRD): Plausible reasoning from observations. Remote Sens. 5, 5999–6025. https://doi.org/10.3390/rs5115999

Mi, C., Huettmann, F., Guo, Y., Han, X., Wen, L., 2017. Why choose Random Forest to predict rare species distribution with few samples in large undersampled areas? Three Asian crane species models provide supporting evidence. PeerJ 5, e2849. https://doi.org/10.7717/peerj.2849

OpenStreetMap Foundation, 2013. OpenStreetMap. Open Database Licens.

Pearce, J., Ferrier, S., 2000. Evaluating the predictive performance of habitat models developed using logistic regression. Ecol. Modell. 133, 225–245. https://doi.org/10.1016/S0304-3800(00)00322-7

Phillips, S., Dudík, M., Schapire, R., 2004. A maximum entropy approach to species distribution modeling. Proc. Twenty-First Int. Conf. Mach. Learn. 655–662. https://doi.org/10.1145/1015330.1015412

Silva, A.C.O., Mello, M.P., Fonseca, L.M.G., 2014. Enhancements to the Bayesian Network for Raster Data (BayNeRD). Proc. Brazilian Symp. GeoInformatics 73–82.

Tarnavsky, E., Grimes, D., Maidment, R., Black, E., Allan, R.P., Stringer, M., Chadwick, R., Kayitakire, F., 2014. Extension of the TAMSAT satellite-based rainfall monitoring over Africa and from 1983 to present. J. Appl. Meteorol. Climatol. 53, 2805–2822. https://doi.org/10.1175/JAMC-D-14-0016.1

Zohmann, M., Immitzer, M., Wöss, M., Gossow, H., Nopp-Mayr, U., 2014. Modelling habitat use of Tetrao urogallus L. in Austria for conservation issues. J. Nat. Conserv. 22, 223–234. https://doi.org/10.1016/j.jnc.2014.01.002
